# T2DM: Why Epigenetics?

**DOI:** 10.1155/2011/647514

**Published:** 2011-11-03

**Authors:** Delphine Fradin, Pierre Bougnères

**Affiliations:** INSERM U986, Bicêtre Hospital, University of Paris-Sud, Le Kremlin-Bicêtre, France

## Abstract

Type 2 Diabetes Mellitus (T2DM) is a metabolic disorder influenced by interactions between genetic and environmental factors. Epigenetics conveys specific environmental influences into phenotypic traits through a variety of mechanisms that are often installed in early life, then persist in differentiated tissues with the power to modulate the expression of many genes, although undergoing time-dependent alterations. There is still no evidence that epigenetics contributes significantly to the causes or transmission of T2DM from one generation to another, thus, to the current environment-driven epidemics, but it has become so likely, as pointed out in this paper, that one can expect an efflorescence of epigenetic knowledge about T2DM in times to come.

## 1. Introduction

The threatened epidemic of T2DM, largely driven by the increase in obesity, is projected to affect >400 million adults worldwide by 2030. Obesity and T2DM, beyond their definition as “diseases,” are becoming the “normal” metabolic fate of a large fraction of modern human populations, notably in those of Asian descent [1]. This human tendency to eat in excess of the needs, to gain fat, and not to have an unlimited insulin secretion capacity, certainly has a widespread genetic background in our species, but the recent epidemics obviously finds its main sources in environmental changes. Those environmental changes can affect phenotype directly or through epigenetic mechanisms that provide an interface with the genome. This is why, in the minds of many, epigenetics has become a leading causative candidate for the causation (and possibly inheritance) of obesity and T2DM. There is no smoke without fire: epigenetic mechanisms have great potential to contribute to the mechanisms and possibly the causes of many environmentally sensitive human diseases. But epigenomics and epigenetic epidemiology are yet at a stage where genomics was 30 years ago, when everyone was working on his part of the puzzle. 

The complete DNA sequence of an organism does not contain the information necessary to specify the organism. The outcome of developmental processes depends both on the genotype and on the temporal sequence of environments in which the organism develops. If the phenotype of the organism of a given genotype is plotted against an environmental variable, the function that is produced is called the *norm of reaction* of the genotype [2]; it is the mapping function of environment into phenotype for that genotype. Since norms of reaction of different genotypes are curves of irregular shape that cross each other, it is not possible to predict the phenotypes of different genotypes in new environments. Indeed, the outcome of development of any genotype is a unique consequence of the interaction between genome and environment. 

The term epigenetics was originally introduced to describe how genetics and environment can interact to give rise to phenotypes during development [3]. Epigenetics more specifically defines cellular modifications that can be heritable during division, but appear unrelated to DNA sequence changes, and can be modified by environmental stimuli [4, 5]. In a more recent view, epigenetics encompasses “mitotically heritable alterations in gene expression potential” [6], a definition that we have favored in this paper. Epigenetic mechanisms typically comprise DNA methylation and histone modifications (referred to as epigenetic *marks*), but also include other mechanisms. Epigenetic marks are established during prenatal and early postnatal development and function throughout life to maintain the diverse gene expression patterns of different cell types within complex organisms but can also arise in mature humans, either by random change or under the influence of the environment. Epigenetic mechanisms, thus, make possible that changes in gene expression in response to an environmental cue can persist in an individual, and possibly in his offspring and grand offspring, long after the cue has disappeared. In addition to mitotic inheritance, some epigenetic marks may be meiotically heritable, conferring the potential for transgenerational epigenetic inheritance [7]. For all of these reasons, a yet hypothetical causal pathway emerges in which some environmental factors that have been increasing in recent decades are commensurately deranging the establishment of epigenetic mechanisms that contribute to plasma glucose regulation, leading to the current explosion in T2DM incidence. Epigenetics might, thus, contribute to self-perpetuate and amplify environmentally sensitive mechanisms through which obesity and T2DM beget obesity and T2DM.

## 2. The Medical Definition of T2DM

The medical definition of T2DM relies upon the minimal value of plasma glucose (PG) that is associated with an increased risk of microvascular and macrovascular complications in late life. The threshold of fasting PG used for defining T2DM has been set at 7.0 mM at the end of the 1990s [8]. People whose fasting PG exceeds this value have, for example, a nearly 2.5 fold increase in coronary disease [9, 10]. This value is also said to represent an optimal cut-off point to separate the components of bimodal frequency distributions of PG, but this cutoff does not always exist or varies across populations [11]. The risk of developing T2DM increases as fasting PG increases, even within the normal range [12–14]. Rarely an isolated condition, T2DM, is most often one of a set of features called metabolic syndrome (MS), of increasing prevalence in middle-aged humans, including obesity, hypertension, dyslipidemia.

## 3. The Causation of T2DM

Observational epidemiology, through population-based cohorts or case-control studies, found a robust association of T2DM with age, obesity, affluent diets, sedentary life, low socioeconomic status (in developed countries), high socioeconomic status (in underdeveloped countries), ethnicity [15], and smoking [16]. Association does not mean causation [17]. However, losing weight, eating less, and exercising are able to reverse T2DM [14], establishing a clear cause-to-effect relationship between these environmental conditions and T2DM. The biological mechanisms through which environmental factors can cause T2DM are partly known for obesity, lack of muscular activity, and ageing [18], but yet remain largely unknown for socioeconomic factors, smoking, and ethnicity. Some of these factors, like adiposity [19], waist-to-hip ratio [20], ethnicity [21], and smoking [22], are themselves determined partly by genetic factors, which are, thus, components of the genetic susceptibility to T2DM [23].

Following enthusiastic claims by geneticists in their early papers, agnostic genome-wide association studies (GWASs) were supposed to provide a novel understanding of T2DM causation. However, the common variants found associated with T2DM provided little biological indication about their implication in T2DM pathogenesis; some were located in gene regions, but functional variants in linkage disequilibrium with the common variants found by GWAS were rarely found [24]. Furthermore, taken together and added, the common variants statistically associated with T2D (each with a small odd ratio) have a very limited capacity to help predict T2DM, compared with simple indices like obesity, waist-to-hip ration, or a familial history of T2DM [25]. It is hoped that new genetic [26, 27] or nongenetic causative factors, like the epigenetic regulation of the expression of genes involved in the maintenance of PG homeostasis, will emerge. 

Since PG is both a predictor of T2DM [13, 14] and the basis for its medical definition, it was attractive to study the variation of PG as a quantitative trait (QT) in the general population. The causality of QT variation such as PG is known to be a mixing of genetic, epigenetic [28], and environmental factors. This turned out to be a poorly fertile approach for T2DM. Environmental factors already associated with T2DM, such as obesity or diet [29], were found causative of variation in PG levels. In contrast, the sharing of genetic factors between T2DM and glucose has remained limited [30–35]. Studying MS was another option to gain understanding in T2DM causality [36], but again the overlap of genetic factors with T2DM was minimal.

## 4. Phenotypic Dissection of T2DM

PG or MS may not be the best phenotypic traits to interrogate in order to understand the causes of T2DM. A phenotypic dissection of T2DM mechanisms at the level of the whole organism, using the tools of physiological investigation, may be more meaningful. T2DM results from the imbalance between glucose production and glucose utilization [37], reflecting the imbalance between the insulin resistance (IR) of muscle, adipose tissue, and liver and the secretion of insulin (IS) by the *β*-cell mass [38–44]. Sometimes called the “endophenotypes” or the “subphenotypes” of T2DM, these QTs are the physiological mechanisms that underlie T2DM [45]. Studying IR and IS separately could overcome the problem that each individual with T2DM may display his own pattern of alterations in IR and IS (phenotypic heterogeneity) [46]. For example, autopsy studies report deficits in *β*-cell mass ranging from 0 to 65% in T2DM [47]. Phenotypic heterogeneity is also observed at a population level; for example, T2DM develops at a younger age in Asian populations than in the European population [1]. Other difficulties are that IR and IS are not easy to measure reliably in hundreds of persons, are dependent on each other (phenotypic interaction), and show various patterns of intra -and interindividual changes during the different life periods preceding T2DM diagnosis [42]. In addition, once T2DM has occurred, it creates its own perturbations including changes in lifestyle, diet, treatment, and metabolic and endocrine dysfunctions which are then difficult to disentangle from primary changes in IR and IS [48]. Three different approaches taken to study the distinctive genetics of IR and IS could inspire future epigenetic studies of T2DM. A first approach is to test the genetic variants found associated with T2DM for their subsequent “sub-association” with IR or IS in nondiabetic individuals [49–53]. Another possibility is to perform GWAS for IR and IS directly in non diabetic individuals [34, 54]. A third approach is to test whether genetic variants in the genes known to be involved in monogenic forms of T2DM play a role in common forms of T2DM [55]. Among susceptibility loci for T2DM identified by GWAS, most are situated near genes involved in IS and almost none in IR [50, 52, 53]. GWASs are usually performed without reference to patients' environment, diet, or physical activity, which have their own effects and may modify genetic predisposition [56]. Since it conveys environmental influences in phenotypic traits, epigenetics are likely to provide new mechanisms to understand the natural history of a failing IS or of augmenting IR in a gene-environment context.

## 5. Complex Traits Inheritance

Distinct from the causation of T2DM, the following two paragraphs deal with the inheritance of T2DM. All causative factors of T2DM are not inherited, while all inherited factors of a disease are necessarily causative, even as a small part of the disease causes. Inheritance, the driving force of evolution, is defined by “the transmission of traits from one generation to another”, while heredity is restricted to “the passing of genetic factors from parent to offspring (or from one generation to the next).” There is no doubt that inheritance in humans includes the four dimensions initially described by Jablonka and Lamb: genetics, epigenetics, learning, and symbols [7]. 

Heritability is the proportion of the phenotypic variance in a population that is attributed to genetic variation between individuals. Phenotypic variation among individuals may be due to genetic, environmental factors, and/or random chance. Heritability analyses estimate the relative contributions of differences in genetic and nongenetic factors to the total phenotypic variance in a population. Heritability estimates are traditionally obtained by comparing the extent of similarity between relatives in classical twin studies, twin-adoption studies, sib/half-sib studies, and transgenerational family studies. Twin studies are unbiased by age effects and help separate environmental from genetic effects. 

Heredity is a part of the T2DM inheritance system, as shown by monozygotic (MZ) twin concordance and familial aggregation [57] but it is further undermined by an impressive degree of “missing heritability” as well. Missing heritability of T2DM comprises the supposedly genetic causes of T2DM that have not yet been identified in current GWASs. Probandwise and pairwise concordance rates for T2DM have been estimated from 0.18 to 0.43 in dizygotic (DZ) twins and from 0.17 to 0.76 in MZ twins [58–62]. If these numbers reflect the true heritability of T2DM, they indicate that inheritance is high for T2DM but has only a limited genetic component. Phenotypic differences within MZ twin pairs are classically attributed to environmental factors. We know now that variation in epigenetic marks between two MZ twins [63–65] can also explain phenotypic differences. MZ twins are derived from the same one-cell zygote, thus, share not only their genomic sequence but also the same initial epigenetic factors except for egg cleavage asymmetry. 

Rather than questioning the true genetic nature of their heritability estimate, geneticists have proposed several explanations for the missing heritability problem [66–68]. First, they took an optimistic look on the idea of rare variants not seen in the current GWASs that would either be in linkage disequilibrium with the observed common variants or have to be found by themselves [69]; few of these rare and functional variants have emerged till now [26, 27]. To explain missing heritability, geneticists also call for the rescue of the concept of gene-gene epistatic interrelations [70] that would increase significantly the role of found gene variants [71, 72], a hypothesis that remains yet impossible to prove but has a lot of biological rationale. Epistatic interactions are not restricted to gene-gene interactions but are widely opened to gene-epigenetic factors or gene-environment interactions. Disregarding gene-environment interactions, as if genes were having their predisposing effects in whatever environment, could also be a key to the missing heritability of T2DM [73–75], given the major role of environmental factors in this disease. As said before, it is also possible that the poor phenotypic resolution of calling T2DM a number of different IR and IS phenotypes, which disregards the phenotypic complexity and heterogeneity of this disease, contributes to the missing heritability problem, by missing not the analysis of the genotype but that of the phenotype [76]. But it also possible that MZ twins, and to a lesser degree DZ twins, or siblings share more nongenetic factors than expected, resulting in an overestimation of heritability, the genetic part of inheritance. Among these nongenetic factors that may confound heritability estimates, one finds again environment in the first place, possibly expressed *via* epigenetic marks inherited from a shared womb or *via *transgenerational epigenetic inheritance of methylation patterns from mother or even grand mother [67]. Epigenetic changes clearly contribute to phenotypes, but the extent to which they contribute to phenotype heritability is unknown. To address this point from a methodological point of view, a study suggested that although epigenetic changes can add to individual disease risk (T2DM causation), they can only contribute to heritability when the stability of methylation transmission during meiosis is very high. To overcome this restriction, Tal et al. have combined a quantitative genetics approach with information about the epigenetic reset between generations and assumptions about environmental induction to estimate the heritable epigenetic variance and epigenetic transmissibility [77].

## 6. Gene-Environment Interactions: The Epigenetic Interface

To state that most complex diseases are caused by an interaction between genome and environment is a cliché. Such interactions, while likely, have for the most part not been demonstrated. Genetics, that is the DNA sequence with its individual variants, is inherited from the parents and will remain intact for the whole lifespan: it will participate to the individual variation of the phenotype, thus, to disease causation, through functional variants that are shared by T2DM cases in excess of controls. Environment is even more complex than genetics, since it is made of a continuous flow of space-time exposures from the time of one-cell zygote to the time of disease onset. A first EWAS (Environmental-Wide association study) was performed in a T2DM cohort and identified potential environmental factors with effect sizes comparable to loci found by GWAS [78]. Since the quantification of environmental influences is notoriously difficult, it is hoped that greater understanding of the epigenome will offer a direct and quantifiable link between putative environmental influences and pathways relevant to T2DM pathogenesis. However, our understanding of environmental influences on epigenetic processes remains rudimentary. Hence, we currently have a limited ability to propose specific environmental exposures whose increasing magnitude might influence epigenetic mechanisms at the population level and thereby contribute to the secular increase in T2DM.

Environmental factors are many. Because of the rapid increase in T2DM incidence in most countries, it is interesting to suspect emerging factors that have become common part of modern human environments. For simplicity, one can distinguish two different parts in our environment: the physical-chemical-biotic world that is surrounding us at every moment of our lives and the psychological exchanges with other human beings. The first world includes available food, climate, nutrients, tobacco, endocrine disruptors [79], food micronutrients such as vitamin D, zinc or folate, trace elements, gut microbiome [80], infectious agents [81], and many other environmental factors that we do not suspect yet to be causative of T2DM. Some of these factors, like food history and agriculture, may have shaped part of the ethnic and geographic differences in T2DM prevalence that are observed across human populations, for example, between European and Asians [82]. But human environment is not restricted to physical, chemical, or biotic factors. “Man is a thinking reed” (B. Pascal, *Pensées, *VI, 346–348). A lot of changes faced by humans, like those defining their lifestyle, mode of feeding, physical activity, and habitat, are directly dependent on personal choices and social interactions, which are themselves dependent on individual behaviors, intellectual skills, education, learning, psychological experiences including stress, as well as emotional, cognitive, and cultural factors. Affluent diets, access to food, feeding choices, and nutrition along whole life belong to both categories of environmental factors since they depend as much on personal choices, social interactions [83], and history [82] as on the foodstuff itself. Gene-environment interactions can also be defined at a population level, where epigenetics is likely to be one of the components of the “ethnical melting pot” [84, 85] that already relies on geographically driven genetic variation [86], possibly fixed in East Asia [87, 88], past adaptations to climate [89], and current environmental and cultural differences: some of these interacting factors are likely to contribute to create variation in T2DM incidence in specific populations [90, 91]. Gene environment interactions should also be viewed in an evolutionary perspective since it is considered that these interactions have shaped the variation of complex traits, among which glucose-insulin homeostasis is central to energy metabolism, thus, human fitness. As a driving force of evolution associated with genetics [92, 93], epigenetics may have played a role in the metabolic adaptation of men to their changing environments.

## 7. The Molecular Bricks of Epigenetics

A person's liver cells, pancreatic *β* cells, muscle cells, adipose cells, and hypothalamic neurons look different, replicate, and function differently, yet they contain the same genetic information. With very few exceptions, the differences between specialized cells are epigenetic not genetic. They are the consequences of events that occurred during the developmental history of each cell type, starting with the initial one-cell zygote and determined which genes are turned on and how their products act and interact (Table 1). Not only specialized cells can maintain their own particular phenotype for long periods, but they can transmit it to daughter cells. This information is transmitted through epigenetic systems. 

**Figure 1:**
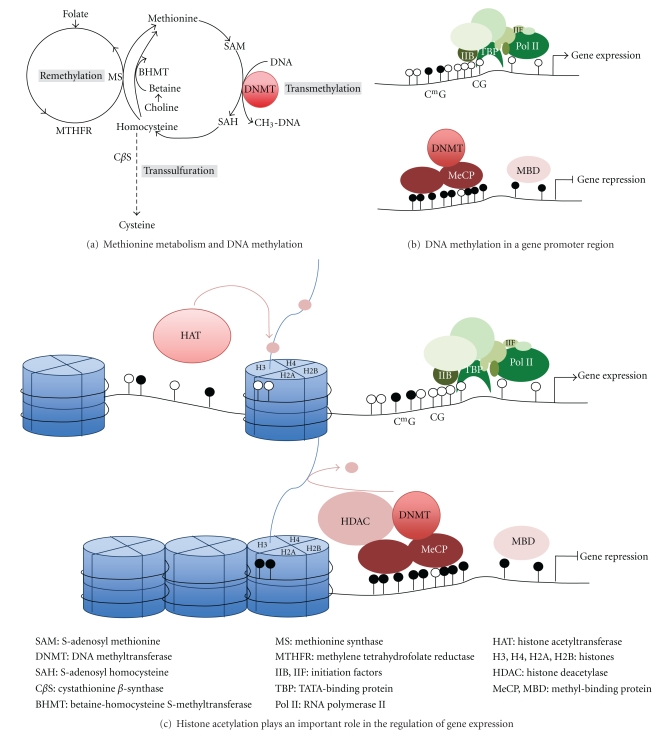
Schematic figure of epigenetic regulation mechanisms.

The first types of epigenetic systems are the chromatin-marking system and the DNA methylation system, which we call “epigenetic *marks*.” Much of today's epigenetic research is converging on the study of covalent and noncovalent modifications of DNA and histone proteins and the mechanisms by which such modifications influence overall chromatin structure. Genomic DNA in eukaryotic cells is packed together with special proteins, termed histones, to form chromatin (Figure 1). The basic building block of chromatin is the nucleosome, which consists of <147 base pairs of DNA wrapped around an octamer of histone proteins composed of an H3-H4 tetramer flanked on either side with an H2A-H2B dimer. Although the core histones are densely packed, their NH_2_-terminal tails can be modified by histone-modifying enzymes, resulting in acetylation, methylation, phosphorylation, sumoylation, or ubiquitination [94]. These modifications are important for determining the accessibility of the DNA to the transcription machinery as well as for replication, recombination, and chromosomal organization. HDACs remove and histone acetyl transferases (HATs) add acetyl groups to the lysine residues on histone tails [94–96]. Although it is well established that HAT activity and increased histone acetylation correlate with increased gene transcription, the exact mechanisms promoting transcription are less clear [97]. Native lysine residues on histone tails contain a positive charge that can bind negatively charged DNA to form a condensed structure with low transcriptional activity. However, different models have recently been proposed, including the histone code hypothesis, where multiple histone modifications act in combination to regulate transcription [97, 98]. Histone methylation can result in either transcriptional activation or inactivation, depending on the degree of methylation and the specific lysine and/or arginine residues modified [99, 100]. Histone methyltransferases and histone demethylases mediate these processes [100]. Chromatin marks are transmitted during cell division and enable states of gene activity or inactivity to be perpetuated. 

One of the main epigenetic systems studied is DNA methylation. DNA methylation occurs principally at a cytosine base, mainly in CpG dinucleotides in vertebrates. Methionine reacts with ATP to form S-adenosyl methionine (SAM), which is the methyl (–CH_3_) donor for DNA methylation (Figure 1). DNA methylation requires the activity of methyltransferases: DNMT1, which copies the DNA methylation pattern between cell generations during replication (maintenance methylation) and DNMT3A and DNMT3B, which are responsible for *de novo* methylation of DNA. The haploid human genome contains approximately 29 million CpGs. The stochastic DNA methylation at particular loci may be altered by environmental exposures and diet and may be heritable transgenerationally [101]. In mammals, centromeric and pericentromeric regions, as well as other repetitive elements are heavily methylated. Many genes also show high degrees of methylation, like bodies of active genes. In contrast most promoter regions and CpG islands (CGIs) lack DNA methylation. CGIs are CpG-rich regions [102], which overlap the promoter region of 60–70% of all human genes [103–107]. Recent studies identified CGI shores as key DNA methylation gene regulatory sites [108, 109]. These regions are defined as regions of lower CpG density that lie in close proximity (≤2 kb) but often not within CGIs. Biophysical studies reveal that DNA methylation plays an important role in repressing accessibility of the transcriptional machinery to the DNA. Indeed, the binding of some transcription factors like Sp1 is known to be methylation sensitive. A second potential mechanism for methylation-induced gene silencing is through its direct binding of specific transcriptional repressors to methylated DNA, like MsecCP-1 and MeCP-2. MeCp-1 binds to DNA containing multiple symmetrically methylated CpG sites [110]. MeCP-2 is more abundant than MeCP-1 and is able to bind a single methylated CpG pair [111]. A third mechanism by which DNA methylation may mediate transcriptional repression is by the recruitment chromatin remodeling enzymes, which change histone posttraductional modification. 

Noncoding RNAs (ncRNAs) are another type of epigenetic actors [112], since they can impact expression of imprinted and nonimprinted genes and are transmitted to daughter cells during mitosis and from sperm and oocyte to the zygote. A large proportion of eukaryotic transcription is bidirectional, producing ncRNAs that can overlap with the transcription of protein-coding genes. NcRNA regulated gene expression by* cis*- and *trans*-acting mechanisms [113]. Some ncRNAs act in concert with components of chromatin and the DNA methylation machinery to establish and/or sustain gene silencing [114]. Through RNA-RNA base pairing, RNA-protein interactions and intrinsic RNA activity, ncRNAs can also regulate RNA processing, mRNA stability, translation, and protein stability and secretion. Some ncRNAs interact with transfer RNAs, ribosomal RNAs, and mRNAs, and can contribute to gene splicing, nucleotide modification protein transport and regulation of gene expression. There are various classes of ncRNAs [115–118]. Two categories have already some relevance in T2DM causation. Micro-RNAs (miRNAs) can regulate gene expression by posttranslational silencing of gene expression and could play a role in T2DM [119–121]. Long noncoding RNAs (lncRNAs) can act as tethers and guides to bind proteins responsible for modifying chromatin and mediate their deposition at specific genomic locations. Large RNAs have been shown to control gene expression from a single locus (Tsix RNA), from chromosomal regions (Air RNA), and from entire chromosomes (roX and Xist RNAs). A gene coding for the lncRNA ANRIL has been found at a locus associated with T2DM in the 9p21.3 region [122]. 

Self-sustaining feedback loops, made of multiple proteins, mRNAs, and ncRNA, is one these systems [7]. Daughter cells can inherit patterns of gene activity present in the parent cell when the control of gene activity involves self-sustaining loops. The initial cue that switched the gene on might have been an external environment change or an internal or regulatory factor. Whatever the cause of the gene being switched on, for as long as the amount of the protein does not fall too much, it will remain active after cell division. The inheritance of the active or inactive state is simply an automatic consequence of more or less asymmetrical cell division. 

A last type of epigenetic system exists as cell membranes, endoplasmic reticulum, and mitochondria membranes, which template the formation of new membranes in daughter cells [93]. 

## 8. Inherited Epigenetic Variations in T2DM

The transmission of epigenetic variants through sexual generations poses theoretical difficulties. The main problem is that the fertilized egg has to be in a state that allows descendant cells to differentiate into all the various cell types. For years, scientists thought that all memories of the “epigenetic past” had to be completely erased before cells can become germ cells, ruling out any possibility that induced epigenetic variations could be inherited. Epigenetics was first suggested by Jablonka to play a role in evolution through Lamarckian inheritance that is a direct modification of the genome by the environment, which is then transmitted transgenerationally [7]. There are currently several routes for inherited epigenetic variation.

### 8.1. Parental Imprinting

The discovery of parental genomic imprinting in the 80s showing that the epigenetic state is not wiped clean was unexpected; some epigenetic information can be passed from a generation to the other. The best known process by which epigenetic marks are transmitted between generations is genomic imprinting, whereby certain genes are expressed in a parent-of-origin-specific manner. Imprinted genes are epigenetically marked and are expressed only from the maternally or the paternally inherited chromosome. They are located in clusters ~1 Mb long. These clusters contain at least one ncRNA that regulates the imprinting of adjacent genes. Genes in these clusters are regulated through DNA sequences known as imprinting control regions (ICRs). ICRs are differentially methylated regions (DMRs) that undergo DNA methylation on only one allele. DNA methylation at these DMRs results in gene repression. Parental imprints are established during gametogenesis and survive the second round of epigenetic reprogramming that occurs during pre-implantation embryo development (Table 1). Imprinted genes have important effects on physiology, brain function, and behaviors by affecting neurodevelopmental processes [123]. Transient neonatal diabetes (TND) is the commonest cause of diabetes presenting in the first week of life. Most patients recover by 3 months of age but could develop T2DM in later life. TND is usually due to genetic or epigenetic aberrations at the 6q24 imprinted locus comprising two genes *PLAGL1 *and *HYMAI *and can be sporadic or inherited. In some individuals, TND may be the initial presentation of a more complex imprinting disorder due to recessive mutations in the *ZFP57 *gene [124]. 

### 8.2. Genetic Variation Inheritance Causing Epigenetic Inheritance

The second type of epigenetic transgenerational inheritance is when obligatory epigenetic variation is dependent on *cis*- or *trans*-acting genetic variation. In these cases, epigenetic variation can be viewed as a readout of the genotype. Substantive evidence for epigenetic heritability has been obtained in age-matched MZ and DZ twin pairs [125–128]. Kaminsky et al. found that MZ twins have more similar DNA methylation patterns than DZ twins across tissues. The most heritable CpG sites were correlated with functional and regulatory regions of the genome, suggesting that more functionally relevant methylation signals are under stronger genetic control. DNA methylation is, thus, a heritable trait on a genome-wide basis, as also shown by recent population-based findings of quantitative trait loci (QTL) for DNA methylation [85, 129, 130], transgenerational and family clustering of methylation patterns [131, 132], and heritable effects of other epigenetic processes [133, 134].

Allele-specific methylation (ASM) is another kind of genetic control on epigenetics whereby DNA methylation is influenced by *cis*-DNA sequence. In loci where it occurs, ASM, may, thus have a major importance in the interpretation of GWAS results. ASM is relatively widespread across the mammalian genome, is quantitative rather than qualitative, and is often heterogeneous across tissues and individuals [135]. DNA methylation is increased on the *FTO* T2DM and obesity susceptibility haplotype, tagged by the rs8050136 risk allele A [136]. Another example of ASM concerns the expression of *NDUFB6* which is decreased in muscle from patients with T2DM. A polymorphism in the promoter of *NDUFB6* (rs629566) is associated with increased DNA methylation on G/G haplotypes and a decline in gene expression in muscle with age [124], suggesting that genetic and epigenetic factors may interact to increase age-dependent susceptibility to IR.

### 8.3. Other Routes of Transgenerational Epigenetic Inheritance

Three different processes of inheritance have been observed in inbred laboratory rodents. The first is *germline epigenetic inheritance*, which occurs when the epigenetic state of the DNA is present in germline cells and is, thus, transmitted to the offspring over many generations. The only solid example we know is the prenatal exposure of pregnant dams to vinclozolin of F_0_ during a sensitive developmental period between days 8 and 15 of pregnancy. This pesticide induces changes in DNA methylation in the first generation (F_1_) of male offspring that persist to the F_4_ generation and beyond in male gametes [137, 138]. Prepregnancy paternal smoking seems capable of inducing epigenetic modifications that pass through the male germline to influence obesity risk in the offspring [139]. Paternal nutrition also matters, since a high-fat diet (HFD) eaten by rat fathers was shown to alter the expression of 642 pancreatic islet genes in adult female F_1_ offspring [140].

Another type of transgenerational epigenetic inheritance could be of major importance to human physiology and diseases. An epigenetic state can affect *parental behavior* in a way that generates the same epigenetic state in offspring [141], so that the maternal care provided by female rats to their young litters leads to the inheritance of their own behavior by their daughters [142]. This effect may persist over many generations. However, if maternal behaviour is altered by stress, there may be an interruption of the transgenerational continuity. 

Another type of epigenetic inheritance concerns alleles that are variably expressed in genetically identical individuals due to epigenetic modifications. In mice, a group of genes, known as *metastable epialleles*, such as Agouti's viable yellow (A^vy^) and A^iapy^ epialleles are sensitive to maternal diet and undergo epigenetic changes during fetal life. They are famous in the epigenetic field because they have allowed the demonstration of true transgenerational inheritance in mice, by transferring embryos between mothers to rule out persistent maternal effects of all kinds [143]. They are not, however, established as “natural” mammalian epialleles, since they have yet been only observed in animals in which a transposon has been inserted upstream of the Agouti coding sequence, leading to overexpression of the Agouti gene and modulation of the mice coat color. In these genetically identical mice, the variation of the Agouti gene expression is strictly dependent on the variation of the epigenetic state at the transposon that determines the phenotype. Maternal methyl-rich or methyl-depleted nutrition is able to modify Agouti's gene expression in offspring, by modifying DNA methylation at this locus. Although metastable epialleles provide an appealing mechanism to epigenetic inheritance [144], there is only nascent indication that they could apply to human diseases. In a single recent study, the methylation patterns of 38 genomic regions were shown to vary independently of genetic variation, across tissues and among individuals in response to environment cues, suggesting that these regions could fit the definition of metastable epiallele [145].

## 9. Noninherited Epigenetic Variations in T2DM

A cause of epigenetic variation that is not inherited from the parents is when alternative epialleles (alleles that can stably exist in more than one epigenetic state) are generated by stochastic events at some finite frequency, regardless of the genotype. Striking examples that are consistent with stochastic alterations in epigenetic marks have been described in somatic cell lineages in humans, including the growing divergence in epigenotype during aging. 

### 9.1. Effects of Environmental Factors on Metabolic Phenotypes and T2DM

#### 9.1.1. The Human Fetus

Interactions between the developing embryo or fetus and its environment can be categorized as developmental plasticity [146], with the aim of producing a phenotype that is matched to the anticipated environment in order to increase the fitness of the organism [147]. There are robust clinical observations [148] in the mid-20th century that early life cues can have lasting effects on metabolic, endocrinem, and neurodevelopmental phenotypes. This was initially reported by Barker in born small offspring of women exposed to poor socioeconomic conditions in South England, Wales, India, and other countries, who have an increased incidence of cardiovascular diseases (CVD) and T2DM when they reach mid-adulthood [149–152]. The relationship between low birth weight (LBW) and later adult diseases gave birth to the “Barker's fetal origins of adult disease hypothesis.” An increased incidence of obesity, T2DM and/or CVD has also been observed in middle-aged adults who had been exposed as fetuses to maternal starving and stress during specific periods of development. Famines and/or wartime [153–155] and maternal infection [156] provided examples suggesting fetal programming, where inappropriate anticipatory choices made *in utero* may underlie the relationship between altered fetal development and the increased metabolic or cardiovascular risks [157], specially when the offspring has to face an affluent postnatal life [155]. 

Persistent changes in DNA methylation may be a common consequence of prenatal exposure to mother's starving. These changes can depend on the gestational timing of the exposure and on the sex of the offspring [158]. Individuals prenatally exposed to the Dutch famine showed decreased methylation of the imprinted *IGF2* gene and increased methylation at *IL10*, *LEP*, *ABCA1*, *GNASAS,* and *MEG3* [159], a finding partly replicated in recently born LBW infants [160]. In rural Gambian women, who experience dramatic seasonal fluctuations in nutritional status, DNA methylation at different metastable epialleles was elevated in offspring conceived during the nutritionally challenged rainy season, providing the first evidence of a permanent, systemic effect of periconceptional environment on human epigenotype. Yet we lack knowledge on mother's signals received by the fetus, and there is no established relationship between these changes in methylation and the occurrence of obesity or T2DM. 

#### 9.1.2. Animal Gestation

In laboratory rodents, maternal nutrition is able to change epigenetic marks during fetal growth, but only a limited number of studies have yet examined DNA methylation changes in a diabetic context. Studies in pregnant rodents subjected to a variety of dietary challenges show a relatively consistent outcome for the offspring, including abnormalities of IS, IR, appetite disturbance, and obesity [161]. In offspring of rat dams given a low-protein diet during pregnancy, which later develop metabolic and cardiovascular abnormalities, there are changes in hepatic expression and in gene promoter methylation and histone acetylation of metabolically relevant receptors, the glucocorticoid receptor (GR) and the peroxisome proliferator-activated receptor *α* (PPAR*α*). These effects are prevented by concurrently supplementing the diet of the pregnant dam with folate, which promotes methyl group provision [162, 163]. Once established, these fetal adaptive responses are not immutable. Metabolic features are particularly apparent when the animals are placed on a high-fat diet after weaning. All of the observed aspects of the induced phenotype after maternal undernutrition are prevented from developing when the female offspring's are treated in the neonatal period with leptin [164, 165]. Leptin administration can give a false developmental cue, signaling adiposity to pups that were actually thin. The pups can, therefore, set their ultimate metabolic phenotype to be more appropriate to a high-nutrition environment. Neonatal leptin treatment not only induces epigenetic and expression changes in specific genes measured in the adult liver, but the direction of these changes is also influenced by previous environmental history (maternal diet). In other studies, growth restriction can also alter histone marks and expression of metabolic genes in offspring, including hepatic *IGF-1 *[166] and *Glut-4 *[167], pancreatic *Pdx1* [142], and hippocampal glucocorticoid receptor (hpGR) [168]. In a primate model of maternal high-fat diet, fetal livers demonstrated increased site-specific histone acetylation and gene expression changes [169]. 

#### 9.1.3. Early Postnatal Life

Adaptation and phenotypic plasticity are not confined to intrauterine life. Early postnatal life is a period of active nutritional changes and the start of social exchanges, mostly with the parents. We have seen before (epigenetic transmission of maternal behavior) that increased pup licking and grooming by rat mothers altered the offspring epigenome at hpGR [168] and at the ERalpha1b promoter [170]. These differences emerged over the first week of life, were reversed with cross-fostering, persisted into adulthood, and were associated with altered histone acetylation and transcription factor (NGFI-A) binding to the hpGR promoter and behavioral responses to stress. Methionine infusion could reverse these effects. Studies of the hippocampal transcriptome identified >900 genes stably regulated by maternal care [171]. Deranging babies' nests, an early-life stress in mice, cause enduring hypersecretion of corticosterone and alterations in offspring's passive stress coping and memory. This phenotype is accompanied by a persistent increase in arginine vasopressin (AVP) expression in postmitotic neurons of the hypothalamus associated with sustained DNA hypomethylation of an important regulatory region that resisted age-related drifts in methylation and centered on those CpG residues that serve as DNA-binding sites for MeCP2. Methylation changes differed widely among the stressed pups (Spengler D., Personal communication), due to different individual perceptions of the stress itself or to the stochasticity of the epigenetic response. Such neurodevelopmental observations may be important for the establishment of early epigenetic effects on metabolic phenotypes in humans, including T2DM. 

#### 9.1.4. Adult Life

Developmental changes continue during the whole life. Exposure to stress continues to be an important environmental cue for triggering persistent epigenetic changes. In adult laboratory mice, social stress can induce long-term demethylation of the corticotrophin-releasing factor* Crf* promoter region [172]. Several studies suggest that the risk of obesity increases by 20% to 50% for several adversities [173] (physical, or verbal abuse, humiliation, neglect, physical punishment, conflict or tension, low parental aspirations or interest in education), but no correlation of these events with epigenetic changes has yet been established, and the database on the role of childhood adversities for the future risk of T2DM or obesity is yet too small to draw conclusions [174]. 

Adult nutrition can also change epigenetic marks and corresponding gene expression in rodents. HFD induces hypermethylation of hepatic glucokinase (*Gck*) gene [175] and induces changes in the methylation patterns of fatty acid synthase (*FASN*) and *NDUFB6* promoters [176]. Anorexia as well as overfeeding can change epigenetic marks at the *POMC* locus [177]. The fact that childhood obesity may be a better predictor of later T2DM than adult obesity may also suggest persistent epigenetic effects of childhood nutrition.

Another mechanism through which nutrition can affect DNA and histone methylation is through the provision of methyl groups used for DNA and histone methylation. A high-fat sucrose diet increases plasma insulin, homocysteine, and methylene-tetrahydrofolate reductase (MTHFR) activity, while decreasing cystathionine-*β*-synthase activity (C*β*S) [178]. Insulin and glucose can affect methionine metabolism [179]. Hyperinsulinemia decreases MTHFR activity [180] and C*β*S-1b promoter activity [181] in hepatocytes; when cells are exposed to elevated insulin and glucose, homocysteine (Hcy) remethylation; hence, intracellular SAM concentrations are increased, due to SAM synthase activity [182]. Elevated glucose further enhances DNA methyltransferase activity that subsequently led to increased global DNA methylation [182]. Obese diabetic rats have increased hepatic C*β*S and betaine-homocysteine S-methyltransferase (BHMT) [183]. Glycine N-methyltransferase knock-out mice have high hepatic SAM levels and hypoglycaemia, suggesting an association between perturbed SAM-dependent transmethylation and abnormal glucose metabolism [184]. Insulin-induced increments of methionine transmethylation, homocysteine transsulfuration, and clearance are impaired in patients with T2DM [185].

### 9.2. Aging of the Organism

Even if early T2DM forms, prompted by childhood obesity epidemics, are emerging, T2DM currently remains a late-onset disease. The effects of passing time are not only made up of a purely temporal biological dimension, but also depend on a steady stream of exposure to various environmental factors. Despite the evidence that DNA methylation is heritable during cell division, substantial changes in methylation patterns take place over time [63, 65, 186–188], suggesting that certain regions [189] of the genome are undergoing epigenetic drift, thus, perhaps contribute to the aging process. We do not know yet the timing of age-related epigenetic changes in the different human tissues. In turn, epigenetic variation can influence cellular lifespan (review in [190]). DNA methylation differences are detectable even between very young MZ twins [64], then epigenetic discordance seems to increase with age [63] in a cross-sectional study which did not assess developmental changes in the same individual. More powerfully, an intraindividual longitudinal study found that global DNA methylation changes >10% over 11 years [131]. The same authors identified later the dynamic VMRs [191], defined by Feinberg et al. as particularly labile sites. Although there is no information about their environmental sensitivity, some of these VMRs could be metastable epialleles. The study of DNA methylation within the promoter region of 3 genes, 5 years apart, in 46 young MZ twins also showed differences in DNA methylation across time [65]. The age of onset of a strongly aged-related disease like T2DM depends on the epigenetic peculiarities of a set of specific genes and the tissues in which these genes are expressed, as well as on environmental and stochastic events. DNA methylation errors that accumulate with increasing age could contribute, for example, by accumulating in the liver or in *β* cells. Such stochastic process taking place over lifetime could be important for causation of T2DM. The liver displays reduced levels of Gck expression in parallel with increased DNA methylation of *Gck* promoter in aged rats [192]. *COX7A1*, which shows decreased expression in diabetic muscle, is also a target of age-related DNA methylation changes [193, 194]. DNA methylation also decreases in *NDUFB6* (cited below) with increasing age [195].

## 10. The Epigenetic Epidemiology of T2DM 

Epigenetic epidemiology provides new opportunities to identify disease biomarkers and to discover links between environmental exposures and diseases. Since a number of thoughtful reviews about genetic epidemiology are available [196–199], we will only discuss the issues of where and when epigenetic marks could or should be studied. There may be separate but coordinated epidemiological approaches for studying epigenetic causation and epigenetic inheritance. Population-based association studies are a way to identify association between epigenetic variation in DNA methylation and disease frequency. Cross-sectional, retrospective case-control or family-based studies are suitable for epidemiological epigenetic studies, as long as the studied samples have a size appropriate to the detection of the expected modest differences in epigenetic patterns. 

DNA methylation is currently the most suitable epigenetic mark for large-scale epidemiological studies, since methyl groups covalently bound to CpG are both durable *in vivo* and survive DNA extraction, unlike histone modifications or ncRNA. This opens the possibility of exploiting existing DNA biobanks. DNA in biobanks is mostly extracted from whole blood, which restricts the meaning and interpretation of the epigenetic observations, given the tissue specificity of most epigenetic patterns. Indeed, the genome contains numerous tissue-specific differentially methylated regions (T-DMRs), defined as a genomic region having a different methylation pattern between tissues. T-DMRs were identified by comparing DNA methylation profiles of various somatic tissues, as well as stem cells and germ cells [109, 200, 201]. For example, in 12 different tissues, 17% of 873 analyzed genes on chromosomes 6, 20, and 22 were found to be differentially methylated in their 5′promoter regions [200]. But many tissues are a mix of different cell types that each shows distinct patterns of DNA methylation. In blood, DNA methylation differs in lymphoid and myeloid cells [202]. For understanding T2DM epigenetics, it would be ideal to obtain information in hypothalamus, *β* cells, liver, muscle, and adipose tissue, but only postmortem tissue can provide valuable insights into the epigenetic profile of such tissues. However, several studies showed that DNA methylation measured in whole blood is a marker for less accessible tissues that are directly involved in disease [203, 204]. Studying changes of metastable allele methylation could allow to avoid part of the problems of tissue specificity [205].

Timing of epigenetic epidemiological studies is another major issue since DNA methylation pattern changes with age, and age increases most of disease risks. The timing of the epigenetic changes is crucial to understanding their role in complex traits. Time-related changes in methylation need to be identified with respect to T2DM onset and progression so as to distinguish between epigenetic changes that could be causal and those that arise secondary to T2DM. To delineate true epigenetic predisposition to T2DM, there is a need to study “baseline” epigenetic profile before T2DM onset, ideally at birth or at the beginning of adulthood, with sampling at regular intervals thereafter. To understand epigenetic predisposition, epidemiologist should, thus, get prepared to the organization of longitudinal studies, for example, in obese populations at risk for T2DM. 

To be credible, the epigenetic hypotheses that can be tested for T2DM should be consistent with both biological mechanisms causing the disease and increased incidence of T2DM. Each hypothetical scenario should involve alterations or individual differences somewhere in a sequence of epigenetic processes [206] comprising (i) a signal which emanates from the environment, (ii) an “epigenetic initiator” which translates this signal to mediate the establishment of a local chromatin context at a given location of the genome, and (iii) an “epigenetic maintainer” which sustains the epigenetic state. 

Introducing epigenetic disease markers in epidemiological studies will face the general Simpson's paradox [207], a statistical phenomenon in which marginal effects, for example, effects associated with a given factor, such as genetic or epigenetic variants can be masked, enhanced, or even reversed in the presence of interactions that are not detected and accounted for [208]. Many interactions may obviously emanate from environment and/or from environmentally sensitive epigenetic processes. The implications of Simpson's paradox for the causality of complex diseases such as T2DM are that it may not be possible to predict a phenotype from a given genotype as long as the interactions among genetic, environmental, and epigenetic components of the system cannot be fully characterized. 

## 11. Conclusion

In conclusion, we have seen that stochastic epigenetic mechanisms can mediate the gene-environment dialog in early life and give rise to persistent epigenetic programming of adult physiology and dysfunctions eventually resulting in T2DM. Understanding how early life experiences can give rise to lasting epigenetic marks conferring increased risk for T2DM, how they are maintained, and how they could be reversed is increasingly becoming a focus of studies in humans. Most often, T2DM is closely linked to obesity, which is itself highly dependent on behavioral, familial, and social interaction [83]. In this respect, epigenetic programming seems particularly important at two levels: (1) the brain, which has a high degree of plasticity and can use epigenetics for the integrated modulation of metabolism and feeding behaviors in response to multiple environmental cues, including nutrition signals and cognitive processes and (2) the metabolic tissues, including the *β* cells, the liver, muscle, and adipose tissue where epigenetic events can allow persistent and time-dependent changes in gene expression potential.

## Figures and Tables

**Table 1:** A glimpse at the epigenetic agenda in the T2DM context.

		Genome	Epigenome	Immediate environment	Known relations with T2D
Germ cells	Oocyte	Meiosis I completed Meiosis II arrested	Establishment of methylation imprints		Maternal diabetes increases oocyte apoptosis
	Spermatozoan		Establishment of methylation imprints Displacement of histones by protamines		

Fecundation					

One-cell zygote to morula	Female DNA	Meiosis II completed	Passive DNA demethylation		
			Imprinted genes retain their germline imprints.		
	Male DNA		Protamines/histones exchange Histone acetylation	Oviductal (maternal)	“Fertility ?”
			Histone monomethylation		
			Active DNA demethylation		
			Methylation remains in centromeric regions, IAP retrotransposons, and paternal imprinted regions		
	Both sex		Histone di- and trimethylation		

Implantation					

Foetus	Embryo XX	PGC female: meiosis I	X inactivation PGC: DNA demethylation and imprint erasure	Placental (maternal)	Maternal T2DM/GDM increases embryo malformations.
	Embryo XY		PGC: DNA demethylation and imprint erasure and then DNA remethylation in prospermatogonia		Maternal nutrition changes DNA methylation on key metabolic genes: *PPAR*α*, IGF2*,…, etc.
	Both sexes		*De novo* DNA methylation Ectoderm (brain), endoderm (liver, *β* cells), mesoderm (skeletal muscle, adipose tissue, blood) Tissue differentiation: T-DMRs		

Birth					

Baby/child	Girl Boy Both sexes		PGC: DNA remethylation — Stochastic modifications	Whole organism	Delivery of a macrosomic fetus. Nutrition affects DNA methylation of key metabolic genes: *FASN, POMC*,…, etc. Insulin and glucose effects on methionine metabolism

Puberty					

Adulthood					

	Both sexes		Aging: stochastic modifications	Whole organism	
